# Clinical and epidemiological features of acute hepatitis D viral in Republic of Moldova

**DOI:** 10.1186/1471-2334-14-S7-O12

**Published:** 2014-10-15

**Authors:** Lilia Cojuhari, Victor Pântea, Gheorghe Placintă, Valentin Cebotarescu, Liviu Iarovoi, Olga Mereuta, Violina Sambris, Elena Ghitu, Irina Gutu, Rodica Tatarciuc

**Affiliations:** 1 Faculty for Continuing Medical Education, Nicolae Testemițanu State Medical and Pharmacy University, Chişinău, Republic of Moldova; 2 Department of Infectious Diseases, Parasitology and Tropical Medicine, Nicolae Testemițanu State Medical and Pharmacy University, Chişinău, Republic of Moldova

## Background

An estimated 10 million people worldwide have dual infection with hepatitis D (HDV) and hepatitis B viruses (HBV). Hepatitis D occurs in populations at risk of hepatitis B virus infection.

## Methods

We studied a group of 26 patients who were hospitalized in the Toma Ciorbă Infectious Diseases Clinical Hospital with acute hepatitis D diagnosis, confirmed by total anti-HDV antibodies and anti-HDV IgM findings using ELISA method and exclusion of other type of hepatitis.

## Results

Acute HDV in both sexes has been observed: women – 7 (26.9%), men – 19 (73.1%). Hepatitis D virus infection occurred through: surgical maneuvers in 2 patients (7.7%), dental – 5(19.2%), sexual – (7.7%), 3 (11.5%) – intrafamilial and undetermined way – 14 (53.9%). The acute onset was in 26 patients (100%), being manifested more frequently in icteric form in 21 (80.8%), than anicteric form – in 5 (19.2%). In 12 patients (46.2%), acute HDV occurs in moderate form and in 14 patients (53.8%) – in severe form. Acute HDV includes asthenic, dyspeptic and arthralgic syndrome. Biochemical investigations: bilirubin – 194.71±31.67 mkmol/L, ALAT – 10.73±0.79 mmol/L/h (p<0.001), thymol – 8.74±1.32 U and prothrombin index – 71.87±2.57%. Hepatomegaly 3.4±0.16 cm – in 100%, splenomegaly 2.0±0.18 cm – in 73.1% patients. Duration of hospitalization constituted 20.73±2.66 days.

## Conclusion

Acute hepatitis D viral affects men more frequently than women, and is manifested through acute onset in the icteric form, severe forms being characterized clinically by the dyspeptic, asthenic, arthralgic and biochemical syndrome through the ALT activity increase, bilirubin, and thymol test. The highest rate of infection was found to be intrafamilial.

